# Ivo Abrahão Nesralla (1938-2020)

**DOI:** 10.21470/1678-9741-2021-0951

**Published:** 2021

**Authors:** Renato A. K. Kalil

**Affiliations:** 1 Department of Surgery, Universidade Federal de Ciências da Saúde de Porto Alegre (UFCSPA), Porto Alegre, Rio Grande do Sul, Brazil.; 2 Post-Graduation Program, Fundação Universitária de Cardiologia, Porto Alegre, Rio Grande do Sul, Brazil.

*“Far better is it to dare mighty things, to win glorious triumphs, even though checkered by failure... than to rank with those poor spirits who neither enjoy nor suffer much, because they live in a gray twilight that knows not victory nor defeat.”*

*Theodore Roosevelt*

“Let's keep going” - this expression, written by hand below the abovementioned quote from T. Roosevelt and left on my desk by Prof. Ivo Nesralla, two days after the death of the first heart transplant patient, at the resumption of transplants in South America (1984), reflects the spirit that guided his life and his legacy. Courage, boldness, and fearlessness associated with charisma, determination, tolerance, resilience, leadership, and the ability to bring together talents and motivate teamwork in search of goals and objectives, motivated only by the ideal that it needed to be achieved, were some of his qualities.

Ivo Nesralla was born in Porto Alegre, in August/18/1938. Son of Nazime and José Nesralla, Lebanese immigrants, he was married to Paula Anita de Mello Nesralla and father of Carlos, Ivo Junior, and Paula, being grandfather of Guilherme, Mariana, Joana, and Vicente.

He joined the Faculdade de Medicina of the Universidade Federal do Rio Grande do Sul (UFRGS) in 1957.

He started his career in cardiac surgery in 1962, as an intern at the Department of Surgery of Santa Casa de Misericórdia de Porto Alegre, then a teaching hospital of the Faculdade de Medicina of UFRGS.

In 1965, as a young cardiovascular surgeon, recently graduated and finishing his training at the former Instituto de Cardiologia do Estado de São Paulo (now Instituto Dante Pazzanese de Cardiologia), Dr. Ivo Nesralla proposed to build a new surgical center dedicated to congenital heart diseases, with resources from a private donation raised by him^[1]^.

With these resources, a foundation was created, and an agreement was signed with the State Health Secretariat to manage the Instituto de Cardiologia (IC). Perhaps the first public-private health partnership in the country was established - the Fundaçao Universitária de Cardiologia (FUC). It opened its doors in 1968 and performed its first cardiac surgery, a pericardiectomy, in May 1969.

In the surgical area, the IC/FUC served 25% of indigent people, a category that included people without resources and without social security coverage, who would only be supported years later by an official social security system. Hospital expenses for these surgeries were borne by the Fundo de Assistência à Cirurgia Cardíaca (FACCA), a philanthropic assistance organization, supported by donations. There was no charge for professional fees by doctors. The President of FACCA was Paula Anita Linck de Mello Nesralla, Prof. Ivo Nesralla’s wife. The institution grew and today it is a hospital with more than 250 beds for Cardiology. It has performed more than 40,000 cardiac surgical procedures and more than 100 heart transplants. It became an internationally recognized Research Center and Higher Education Institution, a status equivalent to Universities, besides being one of the teaching hospitals of the Universidade Federal de Ciências da Saúde de Porto Alegre (UFCSPA), with technical education, undergraduate, medical residency, post-graduate, and post-doctorate. Since 1988, it has a recognized Post-Graduation Program and a research structure responsible for several pioneer achievements, such as the first clinical trial of gene therapy conducted in Latin America. Other examples of pioneer achievements in the assistance area were the first cardiac intensive care unit, the first surgeries on neonates, surgeries on coronary arteries, the first coronary angioplasties, heart transplants, the first left ventricular assistance device implanted in Brazil (1999), first arrhythmia surgeries, and first robotic cardiac surgery in South America (2000)^[2-4]^.

Dr. Ivo Nesralla held the position of chief executive officer of IC/FUC of Rio Grande do Sul (Figure 1) from 1993 to 2017.

**Fig. 1:**
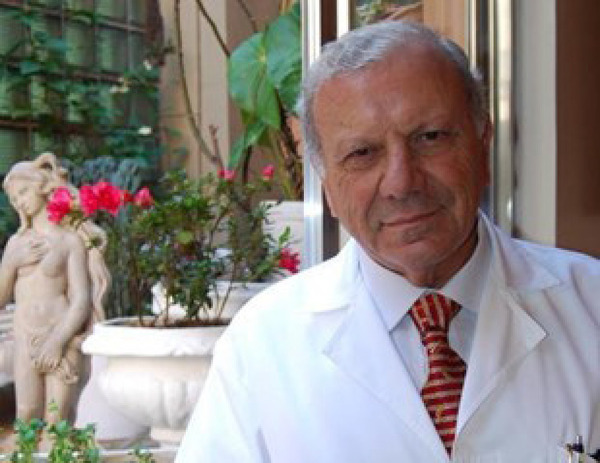
Prof. Ivo Nesralla next to the window of his office at the Instituto de Cardiologia of Rio Grande do Sul/Fundação Universitária de Cardiologia.

In 1969, he assumed as Head of the Cardiovascular Surgery Service of UFRGS Surgery Department, while concurrently put into operation the Cardiovascular Surgery Service of IC/FUC of Rio Grande do Sul.

In 1976, Prof. Ivo Nesralla received the titles of Full Professor and Doctor in Sciences, through public exams and titles. In that year, he assumed the Regency of the Discipline of Cardiovascular Surgery at the Department of Surgery of UFRGS.

In December 1981, he was invited to organize the Cardiac Surgery Service at the Hospital de Clínicas de Porto Alegre, opening it in March 1982.

In December 1986, Prof. Ivo Nesralla held a public exam selection to fill a vacancy for Full Professor of Surgery, in the Department of Surgery of UFRGS.

## MEDICAL SOCIETIES, POSITIONS, TRIBUTES

Prof. Ivo Nesralla had an active participation in contributing for developing the Brazilian cardiac specialty. He was one of the founders and Director-Secretary in the first board of the Sociedade Brasileira de Cirurgia Cardiovascular (SBCCV), in the 1983-1985 term, assuming the Presidency in the 1985-1988 term. He actively participated in all annual SBCCV congresses and in its most significant initiatives during his professional life. He was a Full Member of the Academia Sul-Rio-Grandense de Medicina and the Academia National de Medicina. In 1988, he was invited to preside the Associação Brasileira de Transplante de Órgãos (ABTO), biennium 1989-1990.

On June 20, 1991, he received the title of Citizen Emeritus of Porto Alegre, awarded by the City Council.

In March 1997, he was elected President of the Academia Sul-Rio-Grandense de Medicina.

On March 21, 2002, he received the Medal Cidade de Porto Alegre from the Mayor of Porto Alegre.

In 2015, he received the Medal of Merit Farroupilha, the greatest distinction conferred by the Legislative Assembly of the State of Rio Grande do Sul.

And in 2016, he was highlighted with the “Academia Award” from the Academia Sul-Rio-Grandense de Medicina for his work and contribution to Medicine.

## SCIENTIFIC PRODUCTION

In 1975, Prof. Ivo Nesralla published the first edition of the book entitled *Cardiopatias Cirúrgicas*; in 1982, the second expanded edition was published and yet a third edition, updated and expanded, was published in 1994, edited by the Byk-Procienx Fund^[5]^.

He participated in the Examining Board of 15 public tenders in other Brazilian universities. He was responsible for the training of more than 40 cardiovascular surgeons, at the level of medical residency and postgraduate studies, who practice in several hospitals and are professors at universities in the state and over the country.

During more than 50 years of medical, academic, and scientific activities, he actively participated in 450 regional, national, and international Medical Congresses. Prof. Ivo Nesralla’s publications are distributed as follows: five books, 13 book chapters, and 515 original papers (457 in national and 84 in international journals).

## ARTS, SPORTS, AND SOCIETY

Prof. Ivo Nesralla was an appreciator of the arts and provided numerous services and publications to the community^[6,7]^. In October 1983, he was invited by the Governor of the State of Rio Grande do Sul to preside over the Fundação Orquestra Sinfônica de Porto Alegre; an entity chaired by him for eight years. In 1985, he received the title of Meritorious Member of the Associação dos Amigos do Museu de Arte do Rio Grande do Sul.

In 1998, he assumed the Presidency of the 2^nd^ Mercosul Visual Arts Biennial held by the Fundação Bienal de Artes Visuais do Mercosul in partnership with the Ministries of Culture, Education, and Foreign Affairs, the Government of the State of Rio Grande do Sul, and the Municipality of Porto Alegre. In August 2000, the Presidency of the 3^rd^ Mercosul Visual Arts Biennial was renewed.

**Fig. 2:**
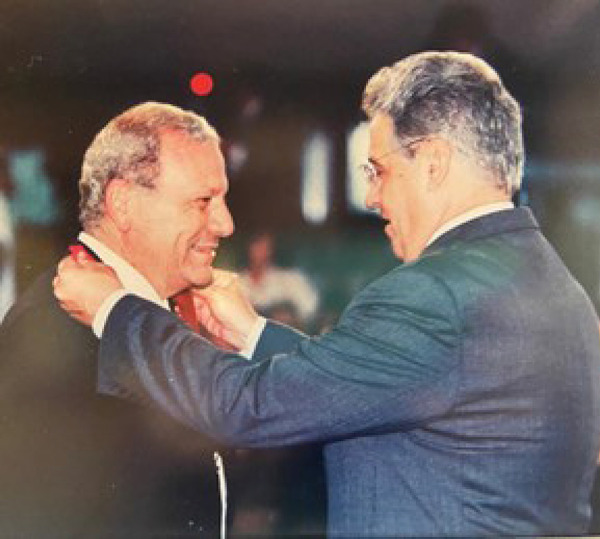
Prof. Ivo Nesralla receiving the Medal of Commander of the Order of Cultural Merit of the Federative Republic of Brazil, by Brazil’s President Fernando Henrique Cardoso.

On November 7, 2001, he received from Brazil’s President, Fernando Henrique Cardoso, a medal of Commander of the Order of Cultural Merit of Brazil (Figure 2).

He was an enthusiast of golf, sport that he practiced twice a week, as long as his health permitted. As such, he presided the Porto Alegre Country Club for two terms, while his wife Paulita presided it for two other periods.

## EPILOGUE

In spite of Prof. Ivo Nesralla’s success in almost all ventures and initiatives, destiny has reserved him some ironies, that this memorial could not be closed without highlighting.

One of them - he performed all types of cardiac surgeries with pleasure, except for pacemaker implants, which he disliked, perhaps traumatized by ancient times when operating with radioscopy without an intensifier in the X-ray room, in very precarious conditions. He did not miss an opportunity to express his distaste for pacemaker implants. Ironically, fate has reserved a cardioverter-defibrillator implant for himself, that has guaranteed his presence among us for a few more years.

The other - in his daily life, he often jokingly expressed that cardiac surgery was not a specialty for women, due to the intensity of the heavy work required, stress, and almost integral dedication, which makes personal and family activities very difficult. But the joke was on him because, among other female surgeons he trained, was his very dear daughter Paula, who was not only trained directly by himself, but also accompanied him in surgical activities while he exercised them and still attended him in the final moments.

Prof. Ivo Nesralla, despite the numerous positions and functions he held, was always attached to the operating room, which he referred to as “the earthly paradise”, where he felt full and very at ease, operating to the sound of classical music, preferably Bach. With the advancement of age and the illnesses that affected him, his presence in the operating room has been decreasing since 2016, but he has always maintained his determination to return. He visited or called the hospital frequently, reminding us to reserve space to operate in the coming weeks. In personal contacts or on the phone, until the week prior to his departure, he always ended the conversation with the expression: “In a few days, when I get better, I will go back and operate with you”.
